# Advanced microscopic evaluation of parallel type I and type II cell deaths induced by multi-functionalized gold nanocages in breast cancer†

**DOI:** 10.1039/c8na00222c

**Published:** 2018-12-10

**Authors:** Sreejith Raveendran, Anindito Sen, Hiromi Ito-Tanaka, Kazunori Kato, Toru Maekawa, D. Sakthi Kumar

**Affiliations:** Bio-Nano Electronics Research Centre, Graduate School of Interdisciplinary New Science, Toyo University 2100, Kujirai, Kawagoe Saitama 350-8585 Japan sakthi@toyo.jp +81 49 234 2502 +81 49 239 1375; JEOL Ltd. 13F, Ohtemachi Nomura Building, 2-1-1 Ohtemachi Chiyoda-Ku Tokyo Japan; Department of Biomedical Engineering, Research Centre for BME, Toyo University 2100, Kujirai, Kawagoe Saitama 350-8585 Japan

## Abstract

Despite aggressive surgical resections and combinatorial chemoradiations, certain highly malignant populations of tumor cells resurrect and metastasize. Mixed-grade cancer cells fail to respond to standard-of-care therapies by developing intrinsic chemoresistance and subsequently result in tumor relapse. Macroautophagy is a membrane trafficking process that underlies drug resistance and tumorigenesis in most breast cancers. Manipulating cellular homeostasis by a combinatorial nanotherapeutic model, one can evaluate the crosstalk between type I and type II cell death and decipher the fate of cancer therapy. Here, we present a multi-strategic approach in cancer targeting to mitigate the autophagic flux with subcellular toxicity *via* lysosome permeation, accompanied by mitochondrial perturbation and apoptosis. In this way, a nanoformulation is developed with a unique blend of a lysosomotropic agent, an immunomodulating sulfated-polysaccharide, an adjuvant chemotherapeutic agent, and a monoclonal antibody as a broad-spectrum complex for combinatorial nanotherapy of all breast cancers. To the best of our knowledge, this manuscript illustrates for the first time the applications of advanced microscopic techniques such as electron tomography, three-dimensional rendering and segmentation of subcellular interactions, and fate of the multifunctional therapeutic gold nanocages specifically targeted toward breast cancer cells.

## Abstract 2

This work evaluates the potential of type I and type II cell deaths in parallel killing of breast cancer cells to mitigate the induced chemoresistance caused.

## Graphical abstract

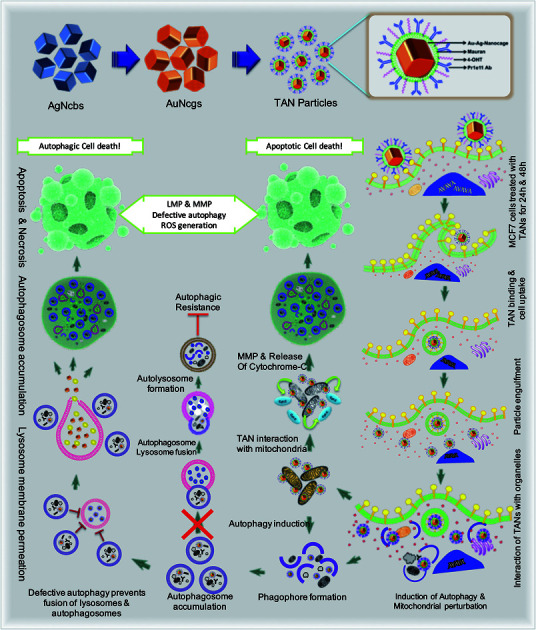

## Introduction

**Fig. 1:**
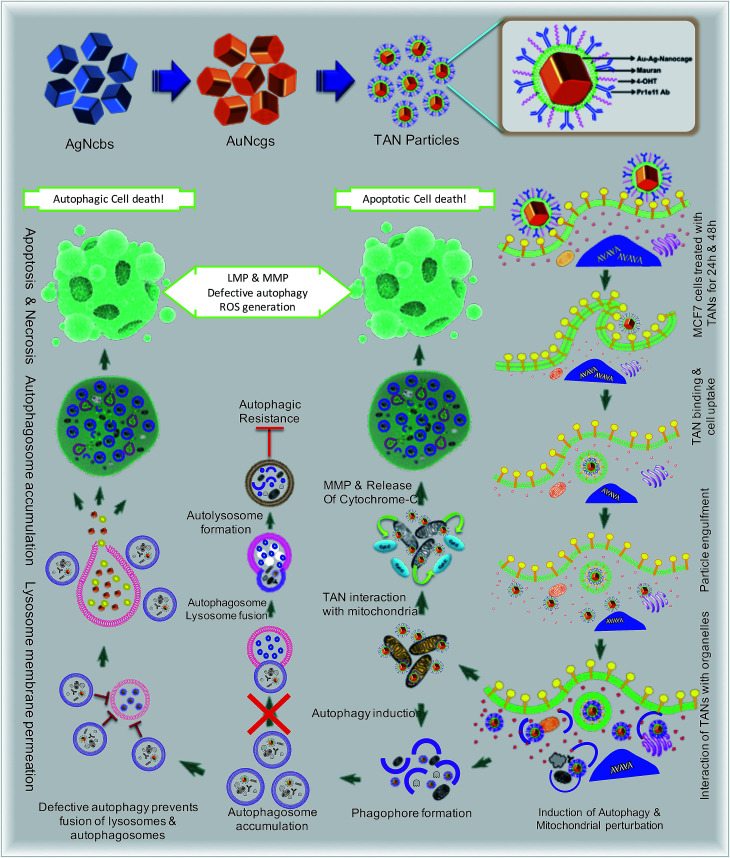
Schematics showing the mechanism of action and effects of TANs in breast cancer cells due to distinct pathways. The representation demonstrates the specific interaction of TANs with subcellular organelles like mitochondria, ER and lysosomes leading to autophagic/apoptotic cell death.

Drug-resistant populations of cancer cells exist despite active chemoradiations and surgical resections.^1,2^ Macroautophagy (hereafter referred to as autophagy) is a major reason for chemotherapy-resistant cancer recurrence and metastasis in many cases.^3–5^ Autophagy is a catabolic membrane trafficking process, by which the cell digests impaired subcellular entities and aberrant bodies.^6,7^ However, in certain cases, autophagy machinery indirectly causes tumorigenesis by inducing resistance against chemotherapeutic agents.^8–12^ Inhibiting drug-induced autophagy by multiple-drug infusions has demonstrated excellent therapeutic benefits, although lethal side effects and toxicities are also observed. To overcome these challenges, strategies comprising combinatorial therapeutic approaches are required. We present a multi-strategic approach to cancer killing by impairing autophagy and parallel induction of apoptosis/necrosis (Fig. 1). This approach involves nano-formulating therapeutic gold nanocages (TANs) encompassing a central gold nanocage (AuNcg) core coated with mauran (MR) polysaccharide, functionalized with 4-hydroxytamoxifen (4OHT) and anti-TROP-2 monoclonal antibodies (MAb).

Bioinert AuNcgs^13^ are biocompatible and optically privileged^14,15^ nanoparticles with impeccable properties and applications in cancer theranostics.^16–20^ Gold nanoparticles have been shown to be lysosomotropic agents that can alkalinize the key subcellular acidic organelle, lysosome.^21^ These suicidal bags regulate cell homeostasis through several mechanisms such as digestion of cell debris, plasma-membrane repair, intracellular signaling for energy metabolism, and so on.^22^ Lysosomal dysfunction results in massive lysosomal membrane permeabilization (LMP), leading to alternative cell death pathways by bypassing autophagic resistance.^23–25^ Inhibition of autophagic flux by preventing autophagosome–lysosome fusion after altering the lysosomal function can lead to autophagic cell death.^21,25^ Destabilization of the lysosomal membrane by nanoparticle toxicity results in the release of hydrolytic enzymes such as cathepsins, cytosolic acidification and reactive oxygen species (ROS), which triggers mitochondrial-mediated intrinsic apoptotic pathways.^23,25^ Mitochondrial perturbation in turn can result in mitochondrial membrane permeabilization (MMP) that seriously injures the cells by triggering several death cascades, resulting in apoptosis/necrosis. Therefore, lysosomes and mitochondria are considered as apparent targets for site-directed cancer therapies.^22,23,26–28^ Here, Pr1E11, a MAb that specifically binds to TROP-2 glycoprotein receptor type I (also known as tumorassociated calcium signal transducer protein; TACSTD2) was used as a target for TAN therapy.^29^ TROP-2 receptors are highly expressed in a variety of carcinomas and epithelial cancers; but less expressed in normal somatic cells. Recently, it was revealed that TROP-2 could be a potential biomarker and an attractive target for drug-MAb therapy against estrogen receptor positive (EsR+) and estrogen receptor negative (EsR−) breast cancers, including the triple-negative subtypes.^30^ Moreover, it was found that 99% of breast cancers have this TACSTD2 expression in its ON state, suggesting it to be a better target for breast cancer therapy. MCF7 cells are recognized as EsR+ and TACSTD2+ breast cancer cells. 4OHT is an active metabolite of tamoxifen and an antagonist of estrogen that binds to EsR receptors. 4OHT was functionalized to MR along with Pr1E11 antibody. MR is an immunomodulating anticancer polysaccharide used as a passivation and functionalization polymer over AuNcgs.^31,32^ With these means, TANs hold a highly promising blend of multiple therapeutic modules in a single nano-entity for a novel combinatorial formulation. Here, we present the subcellular actions of TANs *viz.* lysosome impairment, autophagic dysfunction and induction of mitochondrial permeation that leads to parallel type I and type II cell death in MCF7 breast cancer cells. We demonstrate the modification of autophagy and subcellular interactions of nanocages leading to severe cellular insult using three-dimensional (3D) tomographic reconstruction and rendering along with specific microscopic studies, immunofluorescence and western blot assays.

## Results and discussion

### Cell binding and internalization of TANs

**Fig. 2:**
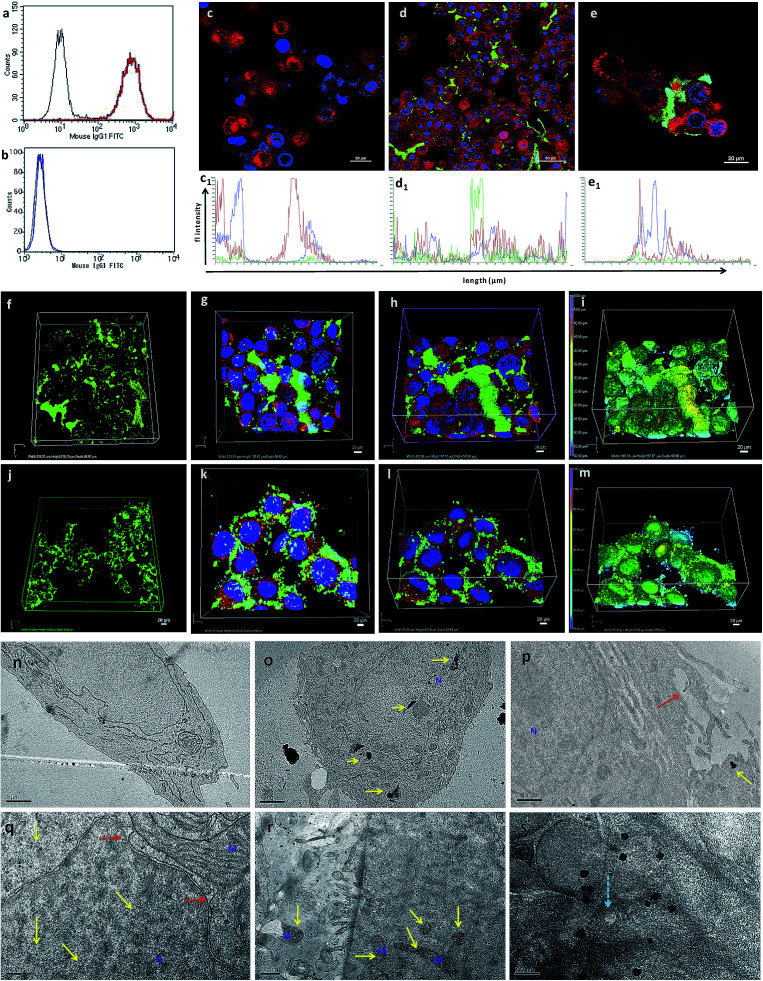
FACS analysis, confocal imaging, and TEM ultrathin section analysis to show the interaction, targeted binding, accumulation and uptake of TANs with breast cancer cells. (a and b) FACS data showing the specificity of free-TROP2 antibody for successful targeting. (a) MCF7 cells (TROP2+ cells). (b) MiaPaca (TROP2-cells). (c) Confocal image of NC cells, without TANs, showing blue (Hoechst) and red (Lysotracker) only. (d) Confocal image of MCF7 cells (TROP2+ and ER+) treated with TANs after 24 h of incubation. Green immunofluorescence (TAN binding) is observed from secondary antibody bound to anti-TROP2 antibody (Pr1E11) present on the surface of TANs interacting with the TROP2 receptor present on the surface of the cancer cells. (e) Magnified image of MCF7 cells showing TAN binding. Corresponding fluorescence intensities of 2D confocal images are also shown (c_1_, d_1_, and e_1_). (f) 3D volume of Z-stacked confocal images obtained from a series of sections, showing the volume of TANs bound to the TROP2 receptors present on the surface of the MCF7 cells by green immunofluorescence. (g) Top view of the Z-stacked volume of MCF7 cells with TAN accumulation. (h) Tilted view of surface rendering of Z-stacked volume of MCF7 cells with TAN accumulation. (i) Topography of the surface rendered image (e) showing the position of nanoparticles in the Z-stack. (j–m) Confocal images of SkBr3 cells (TROP2+ and ER−) showing the broad targeting range of TANs irrespective of ER, PR or HER expressions. (j) 3D volume of Z-stacked confocal images obtained from a series of sections, showing the volume of TANs bound to the TROP2 receptors present on the surface of SkBr3 cells. SkBr3 possess over expression of TROP2 receptor proteins. (k) Top view of the Z-stacked volume of SkBr3 cells with TAN accumulation. (l) Tilted view of the surface rendering of the Z-stacked volume of SkBr3 cells with TAN accumulation. (m) Topography of the surface rendered image (r) showing the position of nanoparticles in the Z-stack. (n) NC cells without vacuolization and organelle damage. (o) MCF7 cells treated with TANs showing accumulation of TANs (high contrast areas shown in yellow arrows) inside nucleus and cytoplasm. Yellow arrows depict the TANs and the red arrow shows the membrane protrusions. (p) Membrane invaginations protruding to engulf nanoparticles. (q) Nuclear invaginations were formed to engulf the nanoparticles and TANs were found inside nucleus. (r) MCF7 cells showing accumulation of TANs inside the cell and interaction with several organelles. (s) Release of TANs from the lysosome compartment (blue arrow). M = mitochondria, N = nucleus.

AuNcgs were synthesized *via* a microwave oven method and were passivated with MR, which was then functionalized with 4OHT and pr1E11 Abs to acquire the hybrid multivalent TANs. The characterization of TANs was performed using high-resolution transmission electron microscopy (HRTEM) (ESI Fig. 1a–e, 2a–k and 3a†), scanning electron microscopy (SEM) (ESI Fig. 1f–i†), dynamic light scattering (DLS) (ESI Fig. 1j†), ultraviolet (UV)-visible spectroscopy (ESI Fig. 3b†) and X-ray photoelectron spectroscopy (XPS) (ESI Fig. 3c–f†). The specificity of the Pr1E11 antibody to TROP2+ cell lines was confirmed with TROP2+ and TROP2− cell lines *via* fluorescence-activated cell sorting (FACS) analysis (Fig. 2a and b). Immunofluorescence was captured using confocal microscopy to ensure the site-specific binding of targeted nanoparticles with TROP2+, MCF7 cells and their cellular uptake (Fig. 2c–e). A 3D volume image shows the collective number of nanoparticles specifically bound to the cell receptors present on the surface of cancer cells as a whole (Fig. 2f–m). Here, SkBr3 (TROP2+, EsR−, HER2+) cells were demonstrated to bind TANs effectively, such as like MCF7 cells (Fig. 2a and b). Thus, TANs demonstrated the specificity to bind to other breast cancer subtypes with TROP2+ and EsR−, which emphasizes the capability of TAN to treat other types of breast cancers irrespective of the common cell receptors studied; EsR, progesterone receptor (PR) and human epidermal growth factor receptor (HER).

Transmission electron microscopy (TEM) sectioning revealed that TAN treatment of MCF7 cells forms elongated cell membrane ruffles and membrane blubbing for acquiring the nanoparticles bound to their surface receptors (Fig. 2o and p). Generally, cells undergo receptor-mediated endocytosis^21^ or macropinocytosis^33^ for engulfing Au nanoparticles (AuNps) and large clusters, respectively.^34^ Negative control (NC) cells do not show any morphological changes, such as protruding cell membrane blubs or abnormal cellular organelles (Fig. 2n). However, TAN-treated cells show nuclear and cell membrane invaginations occurring due to the specific interaction of nano-cargo after receptor-mediated endocytosis (Fig. 2o and p). Most of the nanoparticles (yellow arrows) are found within the cell cytoplasm; nevertheless, a few were also found inside the nucleus. Nuclear entry of TANs over 70 nm in size can be explained unambiguously by the TEM data obtained (Fig. 2d). Functionalization and design of nanoparticles based on saccharide-containing polymer units can mimic the natural process of transporting glycosylated proteins from the endoplasmic reticulum (ER) to the nucleus, thus increasing the chances of nuclear localization by membrane invaginations. Here, MR functionalization could probably mimic similar functions that could enhance herniation or the entrapment of nanoparticles by nuclear membrane invaginations.^35,36^

Internalized TANs were clustered over the cell cytoplasm (Fig. 2q and r) and interact with several organelles leading to the alteration of cellular homeostasis. TEM data show the accumulation of TANs throughout the cell, with specific interactions occurring with mitochondria and lysosome vesicles (Fig. 2s). The tendency of TANs to specifically interact with mitochondria could be attributed to EsR receptors present on the mitochondrial surface, which can harbor the drug, 4OHT.^37^ 4OHT is the active metabolite of the selective estrogen receptor modulator (SERM), tamoxifen, that has been used for the adjuvant therapy of EsR+ breast cancers. Interaction of 4OHT with mitochondria induces mitochondrial-mediated apoptosis and release of cytochrome C (*CytC*) along with several proapoptotic proteins such as caspases.^38^ Similarly, lysosome destabilization by TANs causes the release of cargo from a defective lysosome (Fig. 2s, blue arrow). Previous studies have shown that AuNps are capable of alkalinizing the lysosomes as well as increasing their sizes.^21^ It was reported that AuNps can cause lysosome alkalinization through dissociation of the V_1_ protein from the lysosome-resident V_0_ protein of vacuolar Hp(V)-ATPase. Additionally, TAN-mediated lysosomal impairment resulting in LMP is further explained with a lysosensor assay later in this work. The effect of TANs in mitochondrial-mediated apoptosis and lysosomal-mediated autophagic dysfunction has been studied to evaluate the anticancer activity of TANs.

### Defective autophagy and impairment of autophagosome–lysosome fusion

**Fig. 3:**
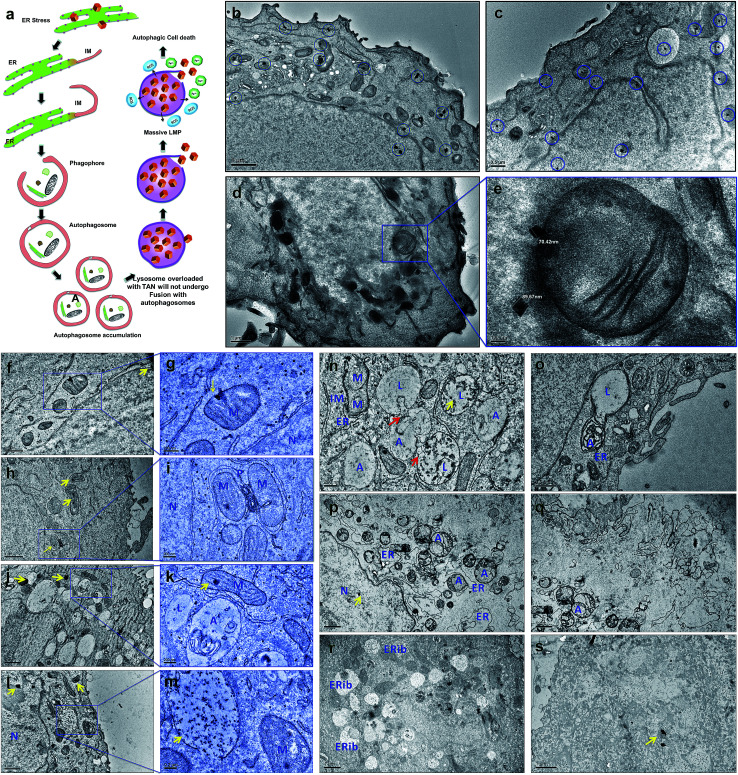
TEM evaluation of subcellular interactions and fate of TANs on MCF7 cells. (a) Schematic representation of TAN interaction with subcellular organelle, ER and initiation of IM, leading to phagophore formation and resulting in defective autophagy. (b) Binding and accumulation of TANs around and within the MCF7 cells respectively. Entrapment of TANs within cytoplasm, nucleus, mitochondria and ER. (c) Many TANs interacting and trapped inside lysosomes and mitochondria, more specifically (highlighted in blue circles). (d) Interaction of TANs with several organelles, especially with mitochondria. (e) Magnified section of image (d) showing the mitochondrial perturbation by TANs. The average size of the TANs entrapped inside the cells was ∼90 nm, which is consistent with the size of the bare nanocages. (f–h) Cellular trafficking of TANs after 24 h treatment with MCF7 cells. (f) Interaction and binding of TAN to mitochondria-nanocage perturbation causes severe damage to outer and inner mitochondrial membrane. (g) Magnified section of image (f) yellow arrows depicts the TANs. (h) Interaction of TANs with various mitochondrial matrix and membrane. (i) Formation of autophagosome by entrapping two damaged mitochondria. A double layered IM or phagophore is surrounding the damaged mitochondria. (j) Vacuolization of cytoplasm and enlargement of lysosomes. (k) Magnified section of image (k) showing the fusion stage of autophagosome and lysosome without TANs. TAN damaged lysosomes are restricted to carry out autolysosome formation as they become damaged by the action of nanocages. (l) Enlarged lysosomes accumulated with several TANs. (m) Magnified section of image (l) showing massive LMP and release of lysosomal content to cytoplasm. This causes the acidification of cytoplasm leading to apoptosis/necrosis. (n–s) Cellular trafficking of TANs after 48 h treatment with MCF7 cells. Red arrows depict the fusion process. (n) Accumulation of damaged lysosomes showing denatured acid phosphatase enzymes inside (dark accumulations). This resulted in defective autophagy leading to massive LMP. (o) Autophagosome-robust lysosome fusion occurred in areas where nanocages were not interacted; however, ER dilation and cytoplasmic blebbing was observed. (p) Enormous accumulation of autophagosomes occur due to the lack of robust lysosome available for fusion, leading to apoptosis/necrosis. Clumping of nuclear materials were also observed. (q) Cell membrane damage and cytoplasmic clearing causes cell death. (r) Concentric ring-structured anomalous mitochondria and ERibs can be seen with clearing cell cytoplasm. (s) Dead cell showing degraded cytoplasm and devoid of cellular organelles. M = mitochondria, N = nucleus, L = lysosome, A = autophagosome, ER = endoplasmic reticulum, IM = isolation membrane, ERib = endoplasmic reticulum inclusion bodies.

Electron micrographic studies of ultrathin sections of TAN-treated MCF7 cells revealed different stages of cells undergoing autophagic, apoptotic or necrotic cell death (Fig. 3). Internalized TANs interact with several organelles in the cell cytoplasm, especially with mitochondria, lysosomes, ER and the nucleus (Fig. 3b–g and ESI Fig. 4†). Induction of autophagy is highly dynamic, and occurs with respect to several physiological stress stimuli. Autophagosome formation involves nucleation, elongation and closure of an isolation membrane (IM) entrapping damaged organelles (Fig. 3a).^39^ IM is generally developed as a double-membranous extension from ER under stress conditions. AuNps exert oxidative stress on ER and mitochondria on internalization.^40^ Thus, IM is formed and detaches from ER to form a phagophore, which matures and engulfs damaged mitochondria, ER and other aberrant bodies present inside the cell (Fig. 3h and n). Here, the cellular ingestion of TANs induces stress on various cell organelles (ESI Fig. 4†) and activates autophagic pathway. Both 4OHT and AuNcgs are previously reported agents that can activate intrinsic autophagic pathways.^8,21,41^ Nevertheless, 4OHT has been studied in terms of activating autophagy, thereby developing chemotherapeutic and anti-estrogen resistance in EsR+ breast cancers.^8^ Thus, blocking of autophagosome function would reduce the emergence of 4OHT therapeutic resistance by facilitating autophagic cell death or type II programmed cell death,^25^ whereas AuNps are identified as potent autophagy inducers despite of their capability to block autophagic flux.^21^ Therefore, a combination of AuNcgs with 4OHT could be a potent nanotherapeutic formulation that could induce autophagic cell death in EsR+ breast cancer cells.

**Fig. 4:**
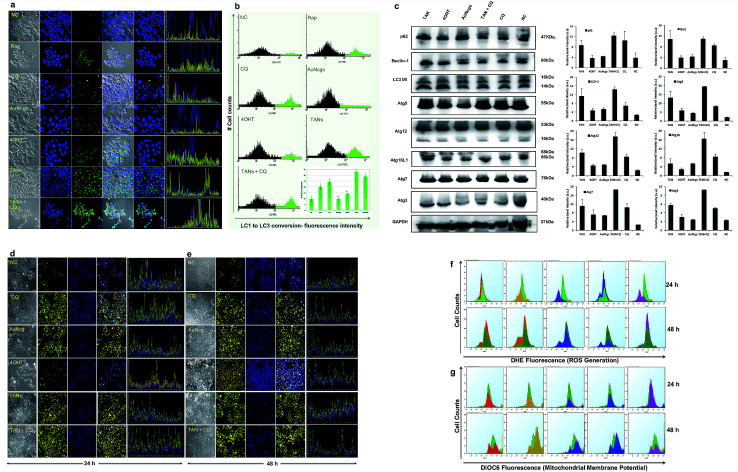
(a–c) Immunofluorescence assay and western immunoblot analysis for autophagy-related proteins (Atg) to show the induction of autophagy. (a) Confocal images showing the induction of autophagy by green fluorescence accumulation. *Cyto* ID green detection reagent accumulates in the perinuclear region, whereas LC3 conversion occurs during autophagy induction. Increased bright green fluorescence is observed during the progression of autophagy, and blue (Hoechst) represents the nucleus. Six sets of test samples were analyzed, *viz.* rapamycin (Rap; 500 nM) (+Ctrl), CQ (20 μM), TAN (32 μM, 100 μl), TAN + CQ (1 : 1, 100 μl), bare AuNcgs (32 μM, 100 μl), 4OHT (0.01 mM) and NC. Autophagy induction was observed by accumulation of green fluorescence in TAN and TAN + CQ-treated cells at a greater extent, which is much higher than the +Ctrl, Rap. Similarly, 4OHT and AuNcgs treated cells also showed green fluorescence, but to a lesser extent. The corresponding fluorescence intensity of the images recorded was also shown to be adjacent to each merged image. (b) FlowCellect autophagy LC3 antibody-based LC3 turnover assay detects the conversion of cytoplasmic LC3I to membranous LC3II by measuring the translocated LC3 in the autophagosomes. The amount of LC3II can be correlated with the number of autophagosomes formed. FACS analysis showed that TANs have the highest capability of LC3I to LC3II conversion, which is 32.9 ± 0.065%, followed by TAN + CQ (∼28.9 ± 2.39%), CQ (∼24.1 ± 2.5%), RAP (∼20.1 ± 2.57%), 4OHT (∼14.2 ± 0.52%), AuNcgs (∼9.7 ± 0.6%) and NC (∼9.6 ± 2.6%). All values were plotted with mean ± standard error of the mean (SE; *n* = 3) and statistical significance was represented with *p* < 0.05 (*) and *p* < 0.01 (**). (c) Formation of autophagosomes involves ubiquitin-like conjugation system with several proteins like *Atg5*, *Atg12*, *Atg16L1*, *Atg7*, *Atg3*, *Beclin-1 (bcl1)*, *LC3A/B*, and *p62 (SQSTM1)*. MCF7 cells were treated with test samples (TAN, 4OHT, AuNcgs, TAN + CQ, CQ and NC) for 24 h and western blotted with respective primary rabbit antibodies and anti-rabbit IgG, HRP-linked secondary antibody from goat. Over expression of Atg proteins and Bcl1 accumulation in TAN-treated cells indicates the induction of autophagy. However, the accumulation of p62 substrate protein shows that TANs direct to defective autophagy by interfering with autolysosome formation, leading to autophagic cell death (type II cell death). Glyceraldehyde 3-phosphate dehydrogenase (GAPDH) served as the loading control. Immunoblot results were analyzed, and relative band intensities were measured and plotted using ImageJ analysis software. All values were plotted with mean ± SE (*n* = 2). (d and e) Immunofluorescence assay showing lysosome impairment, following alkalinization by TANs. (d and e) Confocal images of lysosome impairment assay performed using Lysosensor DND-160 Yellow/blue dye. MCF7 cells were treated with test samples (TAN, 4OHT, AuNcgs, TAN + CQ, CQ (+ctrl) and NC) for 24–48 h and at the end of incubation, cells were stained with Lysosensor DND-160 followed by confocal imaging. NC and 4OHT showed a punctate pattern of yellow fluorescence depicting normal lysosomes, whereas other test samples showed an eccentric pattern of yellow fluorescence throughout the cytoplasmic region due to cytosolic acidification by damaged lysosomes. Results were consistent with both 24 (d) and 48 h (e) test samples. (f) FACS measurement of ROS generated using dihydroethidium (DHE, 5 μM) fluorescence. Cancer cells treated with test samples, *viz.* TAN (red), 4OHT (yellow), AuNcgs (purple), TAN + CQ (blue), CQ (magenta) and NC (green) for 24–48 h were analyzed for ROS generation after DHE staining. All test samples showed ROS generation in comparison with NC, with a sub-G0 peak showing an apoptotic population as well. (g) FACS measurement of Δ*Ψ*_m_ by 3,3-dihexyloxacarbocyanine iodide (DiOC6) staining. MCF7 cells treated with test samples were incubated for 24–48 h and treated with DiOC6 (5 μM) for evaluation change in Δ*Ψ*_m_ the due to *CytC* release. Decrease in the Δ*Ψ*_m_ was observed for all test samples in comparison with NC, however a slight increase in Δ*Ψ*_m_ was observed for 4OHT at 48 h. Irrespective of that it was observed that TAN and other test samples were causing mitochondrial damage by altering the Δ*Ψ*_m_.

Following a lethal cell injury, a series of morphological changes can be observed, including cytoplasmic vacuolization and lysosomes dilation that will lead to apoptosis and necrosis.^42^ TAN ingestion results in the accumulation of enlarged lysosomes (Fig. 3j), which is a characteristic of cells undergoing defective autophagy, lysosome dysfunction and lethal damage.^43^ TEM data reveal matured autophagosomes enclosed with damaged ER and mitochondria, preparing for fusion with lysosomes (Fig. 3k). However, following membrane abrasion and lysosome alkalinization, a massive LMP was observed with lysosomes overloaded with TANs (Fig. 4l and m). This can be attributed to the TAN toxicity, exerted by the AuNcg core on lysosomes, leading to the release of lysosomal cargo into the cell cytoplasm. LMP is a recognized cell death mechanism, and can be partial or massive in nature.^21^ Partial LMP is associated with ROS generation and apoptosis, whereas massive LMP results in cytosolic acidification and necrosis.^25^ This is due to the hydrolytic enzymes, lysosomal-iron and cathepsins released during the process. The associated toxicity exerted by TANs results in lysosome dysfunction and degradation with blockade of autophagic flux. Previous studies have shown that AuNps have the potential to alkalinize lysosomes and cause lysosome impairment.^21^ Autophagosome accumulation demonstrates the blockade of autophagic flux and defective autophagy. A complete destabilization of the cell membrane with apoptotic bodies was observed with cytoplasmic blebs (Fig. 3q). Several dilated ERs with ER inclusion bodies (ERib) were seen, which is a characteristic of defective autophagy and cell death (Fig. 3r and s).^42,44^ Finally, complete necrosis with cytoplasmic shrinkage and destabilized nuclear membrane shows the effect of TANs in the induction of defective autophagy and lysosomal dysfunction.

### Blockade of autophagic flux and autophagic cell death

Induction of autophagy and accumulation of the LC3-II marker protein was evaluated by LC3 turnover assay (Fig. 4a and b). The assay identifies the ability of TAN to induce autophagy by increased accumulation of autophagosomes and/or by blocking autophagosomal maturation to complete the pathway. Among all tested samples, treatment of AuNcgs showed only basal activity of LC3 conversion, even with its *bona fide* ability to impair lysosomes.^21^ The balance between LC3-II production and degradation determines the total amount of LC3-II present. LC3-II turnover can occur under normal conditions, unless otherwise blocked by any lysosome inhibitors such as chloroquine (CQ). CQ is generally used to study the LC3 turnover under blocked autophagy conditions. A combination of TAN + CQ showed increased accumulation of LC3-II protein, similar to TAN alone. This is due to a blockage of autophagosome–lysosome fusion by hampering the function of the lysosome using CQ. Interestingly, cells treated with TAN alone showed much higher LC3-II accumulation, indicating the inhibition of LC3 turnover *via* autolysosome formation. This clearly demonstrates the ability of TAN to disrupt the autophagy pathway *via* lysosome disintegration without any external lysosomotropic agent like CQ. The percentage of LC3 turnover and autophagosome formation was evaluated by FACS analysis (Fig. 4b). Autophagy induced by free drug, 4OHT, progresses with autophagic flux, and finally results in autolysosome formation inducing resistance to cancer cells. 4OHT showed a much lower percentage of LC3-II conversion compared with Rap and CQ, but higher than AuNcgs and NC. Previously, 4OHT was reported to induce autophagy-mediated chemotherapeutic resistance in EsR+ breast cancers^8^ and autophagic cell death in EsR− cancers.^45^ This has been overcome by nanodrug delivery using TANs by inducing autophagic cell death. Activation of autophagy-related genes and production of several associated proteins was evaluated using western blot analysis (Fig. 4c). Overexpression of *Bcl1* and other *Atg* genes shows the successful activation of the autophagy process in TAN-treated cells, which is significantly comparative with 4OHT, AuNcgs and TAN + CQ-treated cells. However, accumulation of *p62* substrate-protein in TAN-treated cells reveals the blockade of autophagic flux and induction of defective autophagy. Increased production of *Bcl1* protein can inhibit tumorigenesis in human breast carcinoma.^46^ Thus, it shows that inducing autophagy and simultaneous impairing the autophagic flux can inhibit the progression of breast cancer and other malignancies.^12,46^

### Lysosome alkalinization and LMP

AuNps are reported to cause lysosome impairment through its alkalinization and enlargement.^21^ Here, we have investigated the action of TANs on lysosomal functions based on pH alterations (Fig. 4d and e). Lysosomal acidification is regulated by acid hydrolases such as acid phosphatase and H+(V)-ATPase enzyme that is associated with membrane ion transport. MCF7 cells treated with test samples show large expanded clusters of yellow fluorescence that denotes increased vacuolization and cytosol acidification.^47^ Cells treated with 4OHT alone show punctuated localization staining with less yellow florescence denoting normal lysosomal function, and are highly comparable with NC, whereas AuNcgs and TAN-treated cells show an eccentric pattern of lysosensor localization with bright yellow fluorescence throughout the cytosol. Lysosomal impairment and massive LMP resulting in cytosolic acidification is directly proportional to the yellow fluorescence observed. Both the test samples, AuNcgs and TANs, showed a similar pattern as observed with the positive control, CQ. CQ is a lysosome inhibitor that causes its enlargement by blocking the enzymatic functions and altering the pH conditions. After 48 h of incubation, yellow fluorescence was increased as result of massive lysosomal leakage. TAN-treated cells were highly comparable with cells treated with TAN + CQ and CQ alone. By this it was evident that treatment of MCF7 cells with TAN alone is highly efficient in lysosome impairment to cause massive LMP. Accumulation of TAN and its biopersistence could be the major reason for lysosomal accumulation and related dysfunction. Increased accumulations of unmetabolized metal nanoparticles containing chemotherapeutic agents are always toxic for normal lysosomal functions and enzyme actions. This will result in autophagic dysfunction, LMP and defective cellular trafficking. LMP increases the presence of several ROS factors and hydrolytic enzymes, which could trigger apoptosis and necrosis.^41^ High concentrations of lysosomal-iron, cathepsin-like enzymes and hydrolases in the cytosol would damage the membrane phospholipids present in the mitochondrial membrane, which in turn could induce MMP. MMP resulting from partial LMP is associated with ROS generation and apoptosis, whereas MMP resulting from massive LMP can result in cytosolic acidification and necrosis.^25^ Thus, this demonstrates that TAN-mediated cell cytotoxicity would cause massive cytosolic acidification that triggers necrosis *via* massive LMP and ROS accumulation.

### ROS generation and mitochondrial membrane depolarization

ROS generation was detected using fluorescing dihydroethidium (DHE) assay. DNA binding was observed by high orange fluorescence, and the amount of fluorescence was measured using FACS analysis (Fig. 4f). The FACS data for DHE intensity measured for different samples based on the amount of ROS generated were compared with the NC. It was observed that TAN interaction induced ROS generation from 24 to 48 h. FACS data demonstrate the formation of a sub-G0/G1 peak in almost all samples when compared with NC, and indicates that the amount of ROS generated within 48 h can induce apoptosis in cancer cells by oxidative damage and other coupled mechanisms. Determination of the cell cycle and percentage of apoptosis induced is discussed later in this paper.

Mitochondrial damage by nanoparticles is recognized as a lethal mechanism for cell deaths *via* apoptosis/necrosis.^27^ Au nanorods with cetyltrimethyl ammonium bromide have previously been reported to induce mitochondria-mediated apoptosis in cancer cells.^48^ Mitochondrial damage can be attributed to the nanoparticle toxicity *via* ROS generation through several means, including direct interaction of nanoparticles with mitochondrial membranes and ROS released as a result of LMP and ER stress. Here, we demonstrate the depolarizing effect of TANs on the inner membrane (InM) and outer membrane (OM) of mitochondria by measuring the Δ*Ψ*_m_. Change in Δ*Ψ*_m_ is considered a crucial parameter to decide the mitochondrial destabilization and MMP. Interaction of TANs and their specific entry to mitochondria has been successfully shown by TEM images (Fig. 3b–e and ESI Fig. 4†). TAN entry to the mitochondrial matrix can be demonstrated with a reduction in matrix Δ*Ψ*_m_. InM permeabilization can be manifested *via* dissipation of the proton gradient responsible for transmembrane potential and OM permeabilization *via* release of membrane proteins such as *CytC*. Mitochondrial depolarization was assessed using lipophilic cationic fluorochrome, DiOC6 (3,3′-tetraethylbenzimidazolylcarbocyanine iodide), which accumulates in the mitochondrial matrix based on the driving force exerted by Δ*Ψ*_m_.^49^ A decrease in the fluorescence intensity or shift in fluorescence emission spectrum is interpreted as an indication of Δ*Ψ*_m_ dissipation (Fig. 4g). Decreased intensity of DiOC6 was measured in case of all test samples except CQ after 24 h of incubation. This indicates a low Δ*Ψ*_m_ when compared with its corresponding NC. OM permeabilization is considered as an important feature associated with permanent loss of Δ*Ψ*_m_. The reduction in Δ*Ψ*_m_ observed is a true indication of the loss of OM integrity. After 48 h of incubation, it was observed that the intensity of DiOC6 fluorescence was further reduced in most of the samples except 4OHT. A drastic increase in Δ*Ψ*_m_ was observed, with 4OHT showing the induction drug resistance causing cell survival.^50^ However, an apoptotic (subG0/G1) peak was observed in all test samples treated for 48 h in the FACS analysis. Thus, reduction in Δ*Ψ*_m_ clearly demonstrates the damage of mitochondria by membrane depolarization on TAN treatment. Depolarization of mitochondrial membrane is responsible for MMP leading to superoxide and peroxide release (ROS generation). This, in turn, leads to mitochondrial-mediated apoptosis and necrosis.

### Electron tomography of mitochondrial perturbation and ER stress

**Fig. 5:**
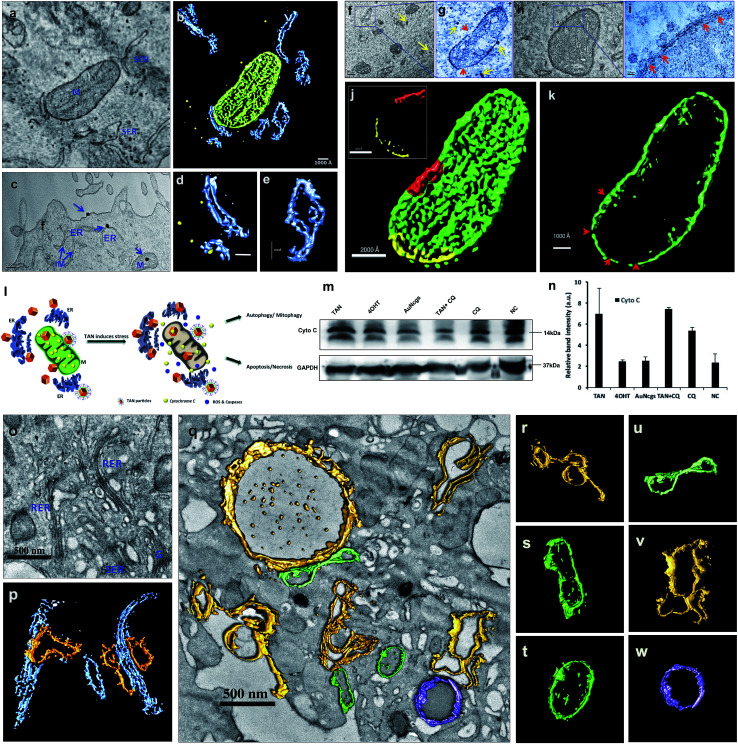
Electron tomographic demonstration of mitochondrial and ER damage by TANs. (a) Ultramicrotomed TEM image of 24 h TAN-treated MCF7 cells showing nanocage interactions with mitochondria and ER. (b) Corresponding tomographic reconstruction and segmentation of mitochondrial and ER interactions with TANs. ER (blue), mitochondria (green) and TANs (yellow). (c) TEM image showing the binding of TANs with cell membrane and their interaction with smooth ER and mitochondria. (d) Segmented tomographic image of ER with nanocage in the cleft. (e) Segmented tomographic image of dilated ER as a result of TAN interaction. Dilation and development of isolation membrane (IM) from ER occurs as a prime step involved in autophagy pathway. (f–i) TEM images of nanocages perturbing mitochondria. It was observed that TANs could bind to outer membrane (OM) of mitochondria and cause serious damage to it. This was explained by measuring the variation in membrane potential and western blot analysis of *CytC* released. Once the particles enter, the matrix becomes damaged and leads to massive mitochondrial membrane permeation (MMP). MMP can generate apoptotic enzymes that in turn induce apoptotic cell death (type I cell death). The yellow arrow depicts nanoparticles, whereas red arrows show the damage caused to the outer membrane (OM) and inner membrane (InM). (j and k) Mitochondrial membrane damage was demonstrated using a segmented tomographic reconstruction of mitochondria. The difference in the thickness of the mitochondrial membrane is shown in the inset of figure (j) along with the abrasions occurred. Scraped membrane regions are shown with red arrows. (l) Schematic representation showing the fate of the cells after mitochondrial and ER damage by TANs. Simultaneous damage to the ER, mitochondria and lysosomes can lead to type I and/or type II cell death in cancer cells. (m and n) Western blot analysis of *CytC* showing an increased release of the membrane protein from TAN-treated cells. GAPDH was used as the loading control. Immunoblot results were analyzed, and relative band intensities were measured and plotted using ImageJ analysis software. All values were plotted as mean ± SE (*n* = 2). M = mitochondria, ER = endoplasmic reticulum, RER = rough endoplasmic reticulum, SER = smooth endoplasmic reticulum, IM = isolation membrane, G = Golgi body. (o and p) TEM image and corresponding tomographic reconstruction of TAN damaged smooth ER (SER) and rough ER (RER). RER (blue) on nanoparticle perturbation develops IM (yellow), which initiates the autophagy pathway (o and p). IM detaches from ER and forms phagophore during this pathway. Most of the SERs are observed with dilated morphology. (q) TEM image showing complete vacuolization and stressed ER with dilated morphology. Dilated ER and autophagosome development (yellow), degrading mitochondria (green) and active intact lysosome (purple). (r) IM developing from stressed ER. (s) Damaged mitochondria with dumbbell shape. (t) Mitochondria with disintegrated cristae and matrix. (u) Dumbbell shaped mitochondria. (v) Autophagosome developing from IM originating from SER. (w) Undamaged intact functional lysosome. M = mitochondria, ER = endoplasmic reticulum, RER = rough endoplasmic reticulum, SER = smooth endoplasmic reticulum, IM = isolation membrane, G = Golgi body.

TEM tomography illustrates the close association of TAN with mitochondrial OM and InM leading to MMP. Similarly, tomographic reconstruction and segmentation of mitochondria revealed the degree of damage observed within OM. The segmented mitochondrial image illustrates differential thickness of the mitochondrial wall and ruptured OM with several fissures (Fig. 5a–k). Toxicity imparted by TAN and its association with mitochondria can be observed as a combined effect offered by 4OHT and AuNcgs together. 4OHT present on the surface of TANs could possibly interact with EsRα and EsRβ receptors present in the mitochondria as they mimic the functions of estrogen. It has been reported that estrogen can enhance the transcription and protein expression of nuclear respiratory factor-1 and upregulates mitochondrial biogenesis in MCF7 cells.^51^ However, the interaction of 4OHT alone with membrane-bound EsR can substitute the function of the estrogen molecules and could increase the transcription of mitochondrial DNA under certain conditions. Nevertheless, TAN-mediated delivery of 4OHT has been demonstrated successfully as being lethal to MCF7 cells by binding and accumulating in the mitochondrial matrix and inducing depolarization as well as MMP. Depolarization of the mitochondrial membrane was further confirmed by the isolation of *CytC* by western blot analysis (Fig. 5m). A significant difference in the amount of *CytC* release was observed from TAN-treated cells compared with other test samples (Fig. 5n). Similarly, AuNps are previously reported as agents capable of causing mitochondrial stress and damage that can lead to apoptosis and necrosis.^27,48^ In addition, TANs impart extreme stress to ER that initiates IM to form phagophore.^52^ Dilated smooth endoplasmic reticulum (SER) and rough endoplasmic reticulum (RER) give rise to double-membrane autophagosomes to engulf damaged subcellular organelles and foreign bodies (Fig. 5o–w and ESI Fig. 6†). It was evident that TANs specifically interact with ER, mitochondria and lysosomes, which relates to type I and type II cell death simultaneously.

### Cell cycle analysis: apoptosis and necrosis

**Fig. 6:**
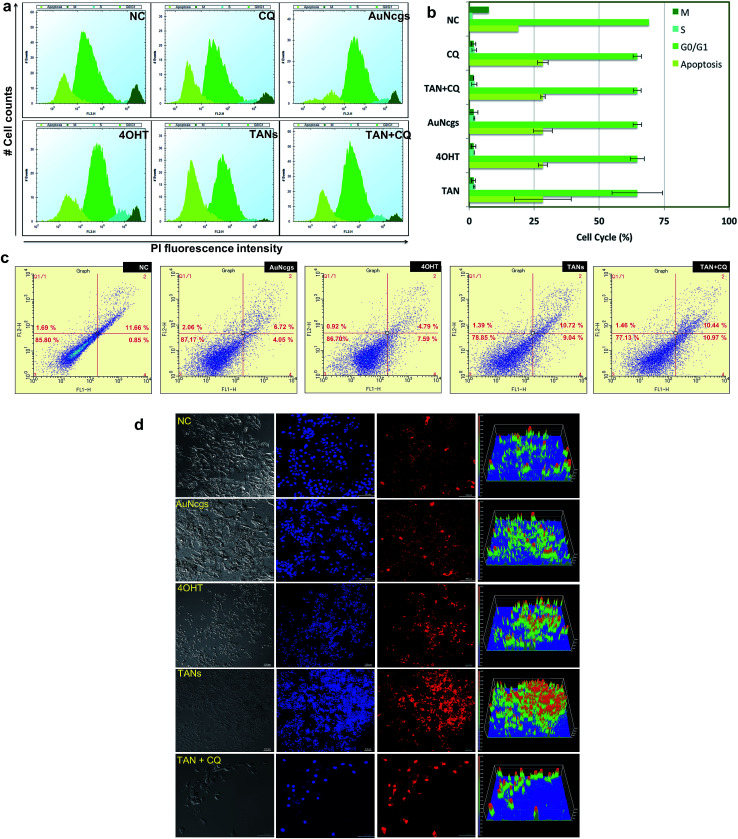
Cell cycle analysis and apoptosis/necrosis assay. (a) MCF7 cells treated for 24 h with various test samples (NC, CQ, AuNcgs, 4OHT, TANs, TAN + CQ) were subjected to cell cycle analysis using PI staining. Cell cycle analysis revealed that TAN-particle-treated cells showed a population under subG0-G1 phase which corresponds to the apoptotic cells. It was evident that apoptosis was higher for TAN-treated cells followed by other test samples. However, AuNcgs treated cells showed less apoptosis compared with other samples. Thus, it reveals the effect of TAN as a combinatorial formulation causing apoptosis in breast cancer cells. (b) Graphical representation of the MCF7 cells undergoing different cell cycle phases as depicted in (a). (c) FACS analysis of MCF7 treated with various test samples for 48 h. TAN and TAN + CQ-treated cells showed a high amount of apoptosis and necrosis. (d) EthD-III staining of MCF7 cells treated with various test samples (NC, AuNcgs, 4OHT, TANs, TAN + CQ) for 48 h, demonstrating necrosis. TAN and TAN + CQ treated cells showed high degree of necrosis compared with other test samples. Corresponding intensity plots for every merged confocal image was shown adjacent to the respective necrotic images.

Apoptosis and necrosis are the two major processes that lead to cell death after lethal cellular injury. Here, we have quantified the cell death by evaluating the content of DNA by cell cycle analysis using propidium iodide (PI) staining (Fig. 6a and b). It was observed that test samples were showing sub-G0/G1 peak, which corresponds to a population undergoing apoptosis in comparison with NC. Similarly, the double diploid or the M-phase for TANs were much less compared with the NC. TAN + CQ showed similar results. However, the cells undergoing M-phase were a little higher than TAN alone. Thus, it was evident that cells treated with TANs undergo serious apoptosis after 48 h of incubation. Similarly, apoptosis and necrosis were further confirmed using ethidium homodimer staining III (EthD-III) and analysis *via* flow cytometry (Fig. 6c) and visualized using confocal microscopy (Fig. 6d). Data observed supported the cell cycle analysis; almost all cells were undergoing apoptosis in comparison with NC. Furthermore, many necrotic cells were also observed with TAN, TAN + CQ, 4OHT and AuNcgs. Confocal microscopy data show that TAN and TAN + CQ displayed massive necrosis compared with NC and AuNcg samples (Fig. 6d). Thus, the ability of TANs to promote efficient cell death *via* apoptosis and necrosis by inducing LMP and MMP was successfully demonstrated.

## Conclusion

Defective autophagy and lysosomal dysfunction are the major emerging mechanisms ascribed to nanomaterial toxicity.^25,40^ This could be a collective result of several other combined toxic effects such as oxidative stress and inflammation induced by nanomaterials. Endocytosis of nanoparticles mostly ends up with lysosome internalization. The lysosomal acidic environment can be extremely hostile for most foreign materials engulfed, except the highly biopersistent nanoparticles such as AuNps.^25^ AuNps are well studied for their ability to activate autophagy, an intrinsic pathway to digest the damaged organelles and aberrant bodies inside the cells *via* lysosomal degradation. In this study, TANs were targeted against TROP2 and EsR receptors present on the surface of MCF7 cells. Generally, cellular ingestion of nanoparticles would increase the interaction of these moieties with cell organelles and induce ROS generation by several mechanisms.^41^ ROS generation will result in ER stress and mitochondrial damage and triggers an autophagic pathway for digesting damaged organelles and proteins. Interestingly, double-targeted AuNcgs become accumulated in lysosome and mitochondria after receptor-mediated endocytosis. TAN exerts severe stress against ER and mitochondria and induces autophagy. Internalization of TANs can induce autophagic pathway *via* a similar mechanism to digest ROS-damaged mitochondria and ER. However, the lysosomal impairment blocks the autophagic flux and leads to autophagic cell death by cytosolic acidification. Similarly, nanoparticle-generated ROS can depolarize the mitochondrial membrane to trigger mitochondria-mediated apoptosis.^25^ Hence ROS generation plays a crucial role in LMP, MMP and autophagy induction in cell death. We investigated the potential of TANs to induce the ROS generation and destabilization of the mitochondrial membrane by altering the membrane potential (Δ*Ψ*_m_). Induction of simultaneous LMP and MMP by TAN can be attributed to two of its major inclusions; AuNcgs^21^ and 4OHT.^12^ Solid AuNps have previously been demonstrated to impart stress over ER, mitochondria and lysosomes.^48^ Here, we have shown the ability of cellular and subcellular-targeted hollow AuNcgs to induce defective autophagy accompanied by massive LMP and MMP with subsequent apoptosis and necrosis leading to type I (apoptotic/necrotic) and/or type II cell death (autophagic cell death).^25,53,54^ Enhanced autophagic activity is generally correlated to metastatic potential and therapeutic resistance in several types of cancers.^10^ However, lysosomal dysfunction and associated toxicity culminating in defective autophagy can be positively steered to establish nanotherapeutic efficiency in cancer treatment. Drugs that act on cathepsins and induce partial or massive LMP are recently investigated as nanotechnological therapeutic targets.^23^ TAN as a single nanodepot holds a promising combination of adjuvant chemotherapeutic agent (4OHT), lysosomotropic agent (AuNcgs), MAb (Anti-TROP2 Ab) and an immunomodulating sulfated polysaccharide (MR) for dual targeted combination therapy of breast cancer. Specificity of TANs to TROP2+ breast cancer cells shows the potential of the TAN prototype nanoformulation as an exclusive hybrid nanoparticle for breast cancer targeting, irrespective of their expression of EsR, PR and HER.^30,55^ Our data suggest that tetravalent inclusions of TAN can lethally target the cancer cell and its subcellular entities leading to necrosis. These findings establish for the first time the possibilities of developing novel nanotherapeutic strategies for inducing several simultaneous killing mechanisms in cancer combination therapeutic strategies using chemically-modified multi-targeted AuNcgs. Future investigations are progressing to unveil the specificity of TAN for other cancers that express TROP2 receptors and the possible death mechanisms involved at the molecular level.

## Author contributions

S. R., T. M., and D. S. K. conceived and designed the experiments. S. R., H. I., and K. K. performed the experiments. S. R., and A. S. performed the TEM tomography, tomographic reconstruction and 3D rendering. S. R., A. S., H. I., K. K., T. M., and D. S. K. analyzed the data. S. R., and D. S. K. wrote the paper.

## Conflicts of interest

The authors declare no competing financial interests.

## Supplementary Material

NA-001-C8NA00222C-s001

NA-001-C8NA00222C-s002

NA-001-C8NA00222C-s003

NA-001-C8NA00222C-s004

NA-001-C8NA00222C-s005

NA-001-C8NA00222C-s006

NA-001-C8NA00222C-s007

NA-001-C8NA00222C-s008

NA-001-C8NA00222C-s009

NA-001-C8NA00222C-s010

NA-001-C8NA00222C-s011
