# Supplementary material for: Advanced microscopic evaluation of parallel type I and type II cell deaths induced by multi-functionalized gold nanocages in breast cancer
Source: Nanoscale Adv. 2018 Dec 10;1(3):989–1001. doi: 10.1039/c8na00222c (PMC9473243; doi:10.1039/c8na00222c)
Supplement: NA-001-C8NA00222C-s003 [file NA-001-C8NA00222C-s003.pdf]

Electronic Supplementary Material (ESI) for Nanoscale Advances.
This journal is © The Royal Society of Chemistry 2018

Advanced Microscopic Evaluation of Parallel Type I and Type II Cell deaths Induced by Multi-functionalized Gold Nanocages in Breast Cancer

Supplementary information
Materials & Methods

1. *Cell culture maintenance*

Breast cancer cell lines were procured from RIKEN Bioresource Centre, Japan and cultured in RPMI-1640 medium supplemented with 10% FBS, 1% L- glutamax, 1% sodium pyruvate and 1% antibiotics-antimycotics. Cells were cultured and maintained until a confluent growth was observed.

2. *Isolation and purification of TROP-2 antibody*

Ascites fluids collected from mouse were used for MAb collection and purification. MAb trap kit (GE Health care # 17-1128-01) was used for purification. Purification protocol was followed according to the product manual. After purification via affinity chromatography, MAb was checked using spectrophotometer at 280 nm for maximum Ab concentration. Higher Ab titres were subjected to dialysis overnight using slide-A-lysis cassettes of pore size 0.45 µm. Filter sterilized MAb was checked for binding specificity with breast cancer cell line and checked using fluorescently tagged secondary Ab (2^0 Ab) isolated from goat. MCF cells were incubated with purified Pr1E11 Ab for 1.5 hr at 4^0C and washed with 2% FBS-PBS to remove unbound Abs. 2^0 Ab (Alexa flour 488- goat anti-mouse IgG- Invitrogen, A11029) specific to Pr1E11 was treated for another 30-45 mins and washed with 2% FBS-PBS. Afterwards, fluorescence was measured using FACS. Pr1E11 Ab isolated was used for functionalizing the AuNcgs for targeting breast cancer cells after determining the specificity of these Abs by FACS.

3. *Synthesis of Therapeutic Au Nanocages (TANs)*

AuNcgs were synthesized via microwave assisted galvanic replacement reaction of AgNcbs as mentioned elsewhere.[1] AgNcbs were used as sacrificial templates for the synthesis of AuNcgs. The process of synthesizing AgNcbs and AuNcgs via microwave heating method is an easy and highly convenient technique as the reaction time has been drastically reduced to several seconds, when compared to conventional methods.[2,3] Washed AuNcgs were morphologically and chemically characterized before and after functionalizing with targeting moieties. MR was extracted from moderately halophilic bacterium *H. maura* as mentioned elsewhere[4]. MR purified via dialysis was lyophilized and powdered MR was used for further studies. Primarily, MR was tagged with 4OHT and Pr1E11. Polyanionic MR possesses anionic functional groups like carboxyl, hydroxyl and sulfate groups, which can be exploited for spontaneous binding of 4OHT and Pr1E11 that has free methyl and amino groups respectively. MR was thoroughly dissolved in deionized water at a concentration of 1mg/5 ml at 4^0C. 4OHT was used as the test drug to adsorb on to MR surface at a concentration of 0.1mM. MR and 4OHT was mixed at 4^0C in dark conditions for few hours and 2 µl of Pr1E11 Ab (1mg/ml concentration) was added to this mixture and allowed overnight incubation under controlled magnetic stirring.

4. *Analyzing AuNcg concentrations using ICP-MS*

Concentration of the AuNcg solutions was measured using Inductively Coupled Plasma Mass Spectrometry (ICP-MS), based on the quantification of Au (III) ions present in various test AuNcg solutions. ICP-MS measurement was performed using iCAP- Q, Thermo Scientific with a plasma Ar flow rate- 15 L/min and a dwell time – 100 ms for each sample. All the samples were microwave (ETHOS UP) digested using 5% aqua regia. A series of Au standard solution were prepared and measured for obtaining a calibration curve, using which the Au (III) concentrations in the test samples were found. As synthesized-AuNcgs were digested using microwave and diluted 100 times using milli-Q-water before ICP-MS measurement. The Au (III) concentration of 100 times diluted as synthesized AuNcg solution was 32 µM.

5. *Characterization of TANs*

AuNcgs were demonstrated to have a negative zeta potential by dynamic light scattering (DLS) measurement. High-resolution transmission electron microscopy (HRTEM) imaging revealed holes in the corners and faces of the AuNcgs (Supplementary Fig. 1a- 1e). Outer shell was approximately 7 nm with an edge length of 70 ± 15 nm (Supplementary Fig. 1f- 1j). AuNcgs were coated with MR. MR is a highly polyanionic polysaccharide consist several negatively charged groups like (SO_4^{2-}, COO^-, PO_4^{2-}) that facilitates strong passivation and binding with Au in AuNcgs. [5,6] Sulfate groups present in MR has high affinity to Au atoms and hence a strong functionalization was achieved (Supplementary Fig. 2). MR was functionalized with 4OHT and Pr1E11 before passivation of the AuNcgs according to the procedure mentioned in the method section. Presence of Sulfur as a coating on the surface of AuNcg shows the successful coating of MR over the cage. Also, the superimposed EDS image (Supplementary Fig. 2k) confirms a slight thicker green layer outside the nanocage, which is from the elemental C from the polymers present in TAN particles. HRTEM characterization and UV-Vis spectra demonstrated the formation of TAN particles with successful passivation of AuNcgs by MR containing 4OHT and Pr1E11 (Supplementary Fig. 3a- 3b). The spectral peak corresponding to the AuNcg in the TAN spectra is considerably lower compared to AuNcgs + 4OHT, which can be due to the complete masking of the cages by the MR polysaccharide during passivation process. TAN formation is further confirmed via XPS analysis by the presence of Au 4f, Ag 3d and S 2p (Supplementary Fig. 3c- 3f). HRTEM, EDS and SAED measurements were carried out using HRTEM (JEM-ARM200F).

6. ***Immunofluorescence for MAb and TAN binding***

Pr1E11, primary mouse monoclonal anitibody was tested for specificity prior to functionalization with AuNcgs using FACS (Fig. 2a & 2b).. MCF7 cells (TROP2+) and MiaPaca (TROP2-) was tested for binding of Pr1E11 ab and then treated with alexa fluor 488- goat anti-mouse IgG (Invitrogen A11029). Fluorescence was detected and compared using FACS- Calibur (BD Immunocytometry Systems, Franklin lakes, NJ). Thus, ensuring the site- specific binding of targeted nanoparticles with TROP2+ cancer cell lines. TAN specific binding and cellular uptake was studied using confocal microscopy, after functionalizing AuNcgs with Pr1E11 and synthesis of TAN particles. MCF7 (EsR+, TROP2+) and SkBr3 (EsR-, TROP2+) cells were treated with TAN particles for 24 hrs and incubated with FITC- labeled anti-mouse IgG secondary antibody after washing twice with PBS buffer. Mitotracker red and hoechst was used to stain mitochondria and nucleus respectively. Cells were visualized using Nikon A1 confocal microscope. TAN bound cells were observed with green fluorescence corresponding to FITC labeled anti-TROP2 Ab, which confirms the specific binding of TANs to TROP2/ EsR receptors present on the surface of MCF7 cells (Fig. 2c-2e). Z-stacking of green immunofluorescent images of TAN particles bound to MCF7 cells were generated to demonstrate the volume of nanoparticles bound to the cell surface at different planes by confocal microscopy (Fig. 2f). 3D stacked volume reveals the intensity of TAN accumulation and collective number of nanoparticles bound to the cell receptors under an enclosed section of imaging. Accumulation of green fluorescence was observed throughout the surface of cancer cells within 24hrs of incubation showing the receptor specificity of TAN binding. Z-volume and isosurface rendering of cells, adhered with TAN particles pinpoints the location of TAN interactions with cells and volume of nanoparticles clustered around them (Fig. 2g- 2i). Furthermore, it was shown that TAN particles could bind to other breast cancer subtypes like SkBr3 (TROP2+ and EsR-), which emphasizes the capability of TAN to treat other types of breast cancers irrespective of the common cell receptor studied, EsR, PR and HER (Fig. 2j-2m).

7. ***Transmission Electron Microscopy:***

MCF7 Cells were treated with TAN particles for 24 and 48 h respectively. 100 μl of the as-synthesized TAN particles were added to the MCF7 cells inoculated in 33mm glass base tissue culture plates. Cells were washed with PBS to remove unbound TANs after incubation and harvested via centrifugation. The pellet size collected should be between 0.5 and 1 mm, which will be >10^6 cells. 1ml of fresh fixation buffer: 2.5% glutaraldehyde in 0.15 cacodylate buffer with 2mM $CaCl_2$ at 35 ^oC (pH 7.4) was added for 2-3 hours in ice. After fixation, cells were washed in cold cacodylate buffer containing 2mM calcium chloride. 1ml of a solution containing 3% potassium ferrocyanide in 0.3M cacodylate buffer with 4mM calcium chloride was combined

with an equal volume of 4% aqueous osmium tetroxide (EMS) and was added to the fixed cells. The tissues were incubated in this solution for an hour, in ice. Cells were washed with ddH_2O at room temperature (RT). Cells were then treated with TCH solution for 20 mins at RT. Tissues were then rinsed again in ddH2O and thereafter placed in 2% osmium tetroxide in ddH20 for 30 minutes, at RT. Following this second exposure to osmium the tissues were washed several times and then placed in 1% Ti blue solution overnight at 4^0C. On the following day, *en bloc* Walton's lead aspartate staining was performed. After adding the lead aspartate solution, the samples were kept at 60^0C for 30 min in the heating oven. The tissues are washed with ddH2O and dehydrated using ice-cold solutions of freshly prepared 20%, 50%, 70%, 90%, 100%, 100% ethanol (anhydrous) respectively for 5 minutes each; then placed in anhydrous ice-cold acetone and left at RT for 10 minutes. Propylene oxide (PO) was added for 15 mins and then it was replaced with PO: Epoxy resin (1:1) mixture, 12-18h at RT. After that the mixture was replaced with pure resin at RT for 6h. Then, the samples were added to respective gelatin tubes and pure resin was infiltrated. Finally, samples were polymerized at 35^0C (6hr) - 45^0C (6Hr) and 60^0C for 1 day in a heating oven[7]. Resin embedded sample blocks were cut into thins sections using Leica EM UC6/MZ6 ultra microtome 300-500 nm sections were obtained on Formvar coated grids for TEM observation. 2D dark field TEM images were captured in JEOL JEM-2100 operating at 200 kV with an Orius CCD camera (1nm pixel size).

8. *LC3 turnover assay*

Assessment of autophagy by electron microscopy is considered as a gold standard in autophagy research.[8] Using TEM, we have successfully demonstrated the blockade of autophagic flux and accumulation of autophagosomes with in the TAN treated cells. Since, the amount of autophagic flux blockade and autophagosome accumulation can only be identified using light microscopy and fluorescence assays, we have used Cyto-ID autophagy detection kit to analyze the amount of autophagosome production based on the intensity of fluorescence generated due to accumulation of the mammalian autophagosome protein. LC3 is a most widely studied autophagosome marker protein, which generally exist as LC3-I in the cell cytoplasm. During the induction of autophagy, LC3-I is conjugated with phosphatidylethanolamine and gets converted in to LC3-II via ubiquitination like enzymatic reaction and gets deposited in the outer and inner membrane of autophagosomes. Unlike LC3-I that remains in cell cytoplasm, LC3-II is associated with autophagosome double membranes and can be detected via green fluorescence through confocal microscopy and flow cytometry. Lysosomal enzymes cleave LC3-II during the downstream process in the autolysosome environment and thus the concentration is decreased. Visualization of endogenous LC3 is crucial for determining the amount of autophagy dysfunction as well as LC3 turnover. Induction of autophagy by TAN particles was studied using Cyto-ID autophagy detection kit (ENZO Life sciences, ENZ-51031). MCF7 cells treated with TAN particles, AuNcgs, 4OHT, CQ, Rap and TAN+ CQ for 24 hrs and compared with negative control. Rap was used as a positive inducer of autophagy and CQ was used as lysosomotropic agent to study the autophagic flux by increasing the pH of lysosomes. Green fluorescence was measured based on the induction of autophagy and accumulation of autophagosomes. Autophagosome detection was carried out by the change of LC3-I protein to LC3-II accumulation in the cytosol. LC3-II accumulation was visualized using confocal microscope and the amount was measured using FACS analysis.

9. *Western blot analysis*

PVDF membrane was blocked with PVDF Blocking Reagent for Can Get Signal ® (TOYOBO) for overnight at 4^oC. Primary antibodies were used as follows; Beclin-1 (1:3000, Cell Signaling Technology ®, #3495), LC3A/B (1:3000, #12741), Atg5 (1:5000, #12994), Atg12 (1:2000, #4180), Atg16L1 (1:2000, #8089), Atg7 (1:2000, #8558), Atg3 (1:2000, #3415), Cytochrome C (1:2000, ab13575), SQSTM1/p62 (1: 2000, ab191852), GAPDH (1:2000, #5174). Primary antibodies were diluted with Can Get Signal ® Solution 1 and incubated for 2 hours at room temperature. Secondary antibodies were used as follows: Mouse TrueBlot® ULTRA: Anti-Mouse Ig HRP (1:5000, ROCKLAND), Anti-rabbit IgG, HRP-linked Antibody (1:5000, #7074). Secondary antibodies were diluted with Can Get Signal ® Solution 2 and incubated for 1 hours at room temperature. All bands were detected using SuperSignal™ West Dura Extended Duration Substrate by ImageQuant LAS 4000. Immunoblot results were analyzed and relative band intensities were measured and plotted using ImageJ analysis software.

10. Lysosome dysfunction and LMP

Lysosomes, typically referred as 'suicidal bags' are single membrane organelles with acidic lumen.[9] They possess several hydrolytic enzymes that mediate degradation of cellular debris and foreign bodies ingested by cells under stress conditions. Lysosomes play critical roles as a promising cancer target or establishing drug resistance in cancer. Lysosomes internalize almost all cancer drugs and nanoparticles initially and decide the fate in their therapeutic regime. However, their self-destruction can be lethal to the entire cellular machinery. To examine the effect of TANs on lysosomes, we have treated the cells with Lysosensor DND160 after incubating the cells with different test samples for 24- 48 hrs respectively. Lysosensor blue/yellow DND-160 is a ratiometric fluorescent probe that helps to identify the acidic cellular organelles especially lysosome based on pH variations. This indicator dye fluoresces in yellow under high acidic conditions and blue under neutral or alkaline environment.

Under normal acidic conditions LysosSensor DND-160 produces specific punctate yellow staining, whereas abnormal acidic organelles stains eccentrically.[10] Yellow and blue fluorescence was visualized using confocal microscopy at an excitation/ emission of 329/440 nm. Fluorescence intensity profiles of the merged images were also recorded.Figure 7a- 7b shows the fluorescent images recorded for MCF-7 cells after incubating with lysosensor probe for specific durations. NC cells show a distinctive punctate pattern of yellow fluorescence indicating a normal state of the cell with uniform distribution of lysosomes.

11. Measurement of ΔΨm and ROS generation

ROS generation and oxidative damage are the major reasons for nanoparticle-mediated cytotoxicity. It can be attributed to several acellular and cellular factors like size, shape, surface charge of nanoparticles and mitochondrial respiration, cell interactions, immune cell activation respectively. ΔΨm and ROS generation was assayed using 3,3'- tetraethylbenzimidazolylcarbocyanine iodide (DiOC6) and dihydroethidium (DHE) stains respectively. DiOC6 is a green fluorescent dye used to determine the change in the mitochondrial membrane potential occurring with respect to the action of test samples. ΔΨm loss was measured using FL1 channel of the HTFC analyzer. Whereas, DHE is one of the commonly used intracellular fluorophore, used to identify the generation of superoxide radicals in cellular ageing studies based on the variation of ROS generation.[11] On oxidation it yields two products, 2-hydroethidium and ethidium. During mitochondrial superoxide generation, they bind to mitochondrial DNA and at higher concentration they can bind to nuclear DNA as well. DNA binding can be observed by high orange fluorescence and the amount of fluorescence was measured using FACS analysis. MCF7 cells treated with test samples were incubated for 24- 48 hrs and washed twice with PBS buffer. Cells were then stained with DiOC6 (5μM) and DHE (5μM) separately and fluorescence intensity was measured using high-throughput flow cytometry (HTFC). Cells treated with DHE were incubated under dark conditions for 30 min and red fluorescence was measured. Fluorescence intensity of test samples was compared with NC to determine the degree of ROS generation.[12]

12. Electron tomographic identification of Mitochondrial damage and ER stress

Electron microscopy is considered most appropriate for cell biology and nanotoxicological studies as they can provide significantly higher special resolution and higher magnifications while imaging associations of subcellular structures with nanoparticles, tissue responses to nanoparticles and necrosis.[13] Similarly, metal nanoparticles like AuNps are high contrast agents that can be easily visualized inside cellular organelles using electron microscopy and tomographic analysis. Electron tomography of the nanoparticle targeted breast cancer cells were performed with ultra-thin sections after incremental tilting of +60 to -60. 2D images captured were aligned using TEMography software and segmentation of the aligned images was performed with Colorist Software. Mitochondria and ER interactions with several TAN particles were subjected to TEM tomographic analysis and reconstruction. It was observed that the nanoparticles interact directly with the OM of mitochondria and clefts present in the ER. The images of mitochondria and ER undergoing stress after TAN treatment was captured and subjected to tomographic reconstruction and segmentation (Fig. 5a- 9k, supplementary Fig. 5). TEM images reveals the specific-nanoparticular toxicity towards the ER and mitochondria after the endocytosis process. TAN breaches the surface of ER and mitochondria and it initiates the process, which was followed by severe cellular insult leading to type I and type II cell deaths (Fig.5c- 5l).

Our present study fortifies the previous reports of nanoparticle toxicity with ER stress (Fig. 5o- 5q) leading to IM formation and autophagy.[14,15] Besides that, OM rupture and mitochondrial penetration of TANs delineate the probability of inducing apoptosis simultaneously over autophagy, as evident from the TEM data (Fig. 5f-5k) and protein blot results (Fig. 5m- 5n).

13. Cell death analysis (PI and EthD-III staining)

Apoptosis is an active programmed process of cell death regulated by cell itself after as series of unfavorable events. Whereas necrosis occurs as a result of serious cellular insult leading to complete destabilization of cells with disintegration of sub-cellular organelles. Cell cycle analysis was performed using PI staining. MCF7 cells treated with test samples were incubated with PI and washed twice to remove excess stain. Later the cells were analyzed using High Throughput flow cytometry (HTFC) for determining the cells undergoing various stages of cell cycle. Necrosis was visualized using ethidium homodimer III staining (EthD-III) with confocal microscopy. EthD-III is a highly positive charged nucleic acid probe used for staining exclusively necrotic cells and is impermeant to live or apoptotic cells. TAN treated necrotic cells were fluoresced in red and visualized using confocal microscopy at 538/617 nm.

14. Statistical analysis

TEM experiments were conducted with multiple ultrathin sections of test samples treated with MCF7 cells. All immunofluorescence assays were performed in duplicates and quantitative data are expressed as the mean ± S.E. Statistical significance comparisons between experimental groups were made using Student's *t*-test. Probability values were represented as follows: $P<0.05$= *; $P<0.01$= **.

References

1 S. Raveendran, A. Sen, T. Maekawa and D. S. Kumar, *Nano Res.*, 2016, **10**, 1078–1091.
2 S. E. Skrabalak, L. Au, X. Li and Y. Xia, *Nat. Protoc.*, 2007, **2**, 2182–2190.
3 Y. Sun and Y. Xia, *Science*, 2002, **298**, 2176–2179.
4 S. Raveendran, V. Palaninathan, N. Chauhan, Y. Sakamoto, Y. Yoshida, T. Maekawa, P. V. Mohanan and D. S. Kumar, *Carbohydr. Polym.*, 2013, **98**, 108–115.
5 S. Raveendran, N. Chauhan, V. Palaninathan, Y. Nagaoka, Y. Yoshida, T. Maekawa and D. S. Kumar, *Part. Part. Syst. Charact.*, 2015, **32**, 54–64.
6 S. Raveendran, A. R. Girija, S. Balasubramanian, T. Ukai, Y. Yoshida, T. Maekawa and D. S. Kumar, *ACS Sustain. Chem. Eng.*, 2014, **2**, 1551–1558.
7 S. Raveendran, V. Palaninathan, Y. Nagaoka, T. Fukuda, S. Iwai, T. Higashi, T. Mizuki, Y. Sakamoto, P. V. Mohanan, T. Maekawa and D. S. Kumar, *Int. J. Biol. Macromol.*, 2015.
8 D. Huang, H. Zhou and J. Gao, *Sci. Rep.*, 2015, **5**, 14361.
9 M. Gyparaki and A. G. Papavassiliou, 2014, **20**, 239–241.
10 S. J. Hurwitz, M. Terashima, N. Mizunuma and C. a Slapak, *Blood*, 1997, **89**, 3745–54.
11 J. Wilhelm, R. Vytasek, I. Ostadalova and L. Vajner, *Mol. Cell. Biochem.*, 2009, **328**, 167–176.
12 Y.-H. Ling, L. Liebes, Y. Zou and R. Perez-Soler, *J. Biol. Chem.*, 2003, **278**, 33714–33723.
13 A. Ostrowski, D. Nordmeyer, A. Boreham, C. Holzhausen, L. Mundhenk, C. Graf, M. C. Meinke, A. Vogt, S. Hadam, J. Lademann, E. Ruhl, U. Alexiev and A. D. Gruber, *Beilstein J. Nanotechnol.*, 2015, **6**, 263–280.
14 E. Panzarini, V. Inguscio, B. Anna Tenuzzo, E. Carata and L. Dini, *Cancers (Basel).*, 2013, **5**, 296–319.
15 S. T. Stern, P. P. Adiseshaiah and R. M. Crist, *Part. Fibre Toxicol.*, 2012, **9**, 20.

Supplementary figures captions

1. **Supplementary Figure 1: Electron microscopic characterization of bare AuNcgs synthesized via microwave technology: a,** high magnification TEM image of bare AuNcg synthesized using microwave method. **b- c,** high magnification image of highlighted area of **a** and its corresponding line profile from the highlighted area of the HRTEM image of AuNcg shown in **b.** Line profile shows the lattice spacing of the Au atoms present in the AuNcgs as 2Å. **d,** reduced fast Fourier Transform (FFT) image of AuNcg shown in **b**. **e,** line profile of FFT image shown in figure **d**. **f,** SEM image of AuNcgs. **g,** EDS mapping image of AuNcgs- mapping image in the inset. **h,** SEM image of AuNcgs with size measurement shown. **i,** size distribution plot for AuNcgs based on SEM measurement data. **j**, cumulative percentage of hydrodynamic size distribution of AuNcgs measured using DLS.

2. **Supplementary Figure 2: Electron microscopic characterization of TAN particles: a,** high magnification TEM image of TAN particle. **b-c,** HRTEM image highlighting the thickness of edge of nanoparticle and corresponding line profile showing the edge thickness (~7 nm). **d-e,** HRTEM image highlighting the thickness of MR coating on nanoparticle and corresponding line profile showing the polymer thickness (~ 1.4 nm) outside the nanoparticle. **f- k,** energy dispersive spectrum (EDS) and mapping of TANs with their constituting elements. **f=** bright field image, **g=** C, **h=** Au, **i=** Ag, **j=** S, and **g=** Merged image of C, Au and S.

3. **Supplementary Figure 3: a,** TEM image of TAN particles. **b**, UV-vis spectra for TAN, AuNcgs+4OHT, 4-OHT, MR and AuNcgs (inset). **c,** XPS wide spectra for TAN particles measured on silicon substrate. **d,** XPS spectra for Au 4f in TAN. **e,** XPS spectra for Ag 3d in TAN. **d,** XPS spectra for S 2p in TAN. UV- vis spectra and XPS spectra reveals the functionalization of MR on the surface of AuNcgs and encapsulation of 4OHT drug to form the TAN particles.

4. **Supplementary Figure 4: TEM images of ultra- thin sections of MCF7 cells treated with TAN particles for 24- 48 hrs: a-e,** MCF7 cell showing abnormal subcellular structures (**a, c**). 1= dumbbell shaped mitochondria (magnified in **b**), 2= swollen and dilated SER, 3= swollen and dilated RER, 4= TAN inside the nuclear matrix, 5= double membrane autophagosome (magnified in **d**), 6= mitochondrial blebbing (magnified in **e**). **f**, TANs bound to cell surface and internalized (highlighted in blue circles). **g**, effect of TAN action on subcellular organelles; LMP can be observed due to breakage of lysosomal membrane and permeation. Also accumulation of autophagosomes can be seen causing autophagic cell death.

5. **Supplementary Figure 5: a-d,** TEM tomographic reconstruction and segmentation of mitochondria and ER of MCF7 cells treated with TAN particles.

6. **Supplementary Figure 6: a,** TEM image showing complete vacuolization and stressed ER with dilated morphology. **b,** IM developed from stressed ER giving rise to double membranous structure to form autophagosome. Segmented reconstruction of phagophore at two different angles. **c,** segmented reconstruction of mature autophagosome at two different angles.

7. **Supplementary movie 1:** Confocal Z-series movie of MCF7cells bound with TAN.

8. **Supplementary movie 2:** Confocal Z-series movie of MCF7cells bound with TAN.

9. **Supplementary movie 3:** Confocal Z-series movie of MCF7cells bound with TAN, z to x-axes.

10. **Supplementary movie 4:** Confocal Z-series movie of SkBr3cells bound with TAN

11. **Supplementary movie 5.** Confocal Z-series movie of SkBr3 cells bound with TAN.

12. **Supplementary movie 6.** TEM tomography movie for MCF7 cells treated with TAN- mitochondrial and ER damage (segmented).

13. **Supplementary movie 7.** TEM tomography movie for MCF7 cells treated with TAN- mitochondrial and ER damage (raw).

14. **Supplementary movie 8.** TEM tomography movie for MCF7 cells treated with TAN- mitochondrial and ER damage (segmented).

15. **Supplementary movie 9.** TEM tomography movie for MCF7 cells treated with TAN- ER damage (raw).

16. **Supplementary movie 10.** TEM tomography movie for MCF7 cells treated with TAN- mitochondrial damage, ER damage, IM and double membrane autophagosome formation (raw).

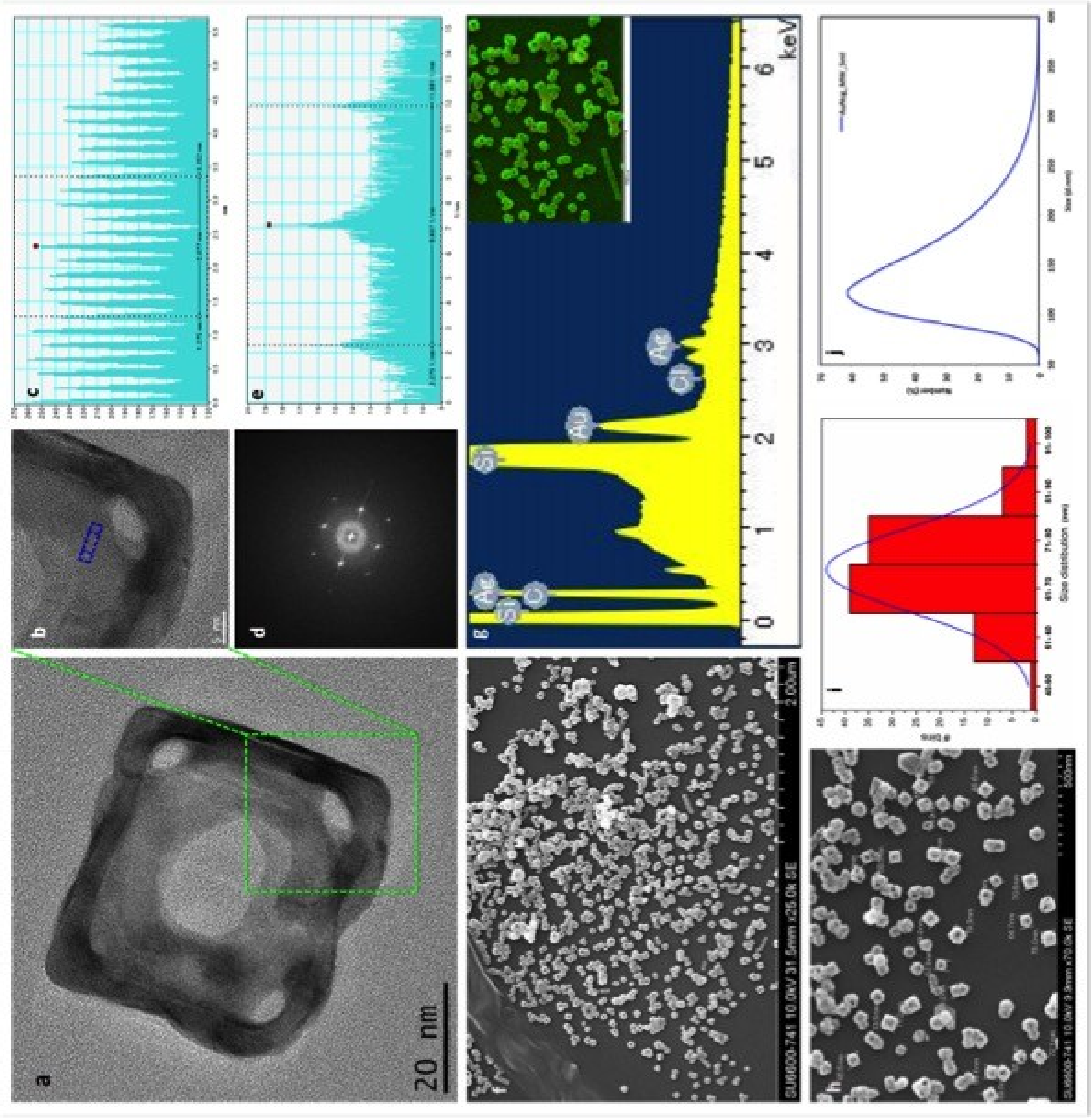
20 nm
keV
Au
Si
Cl
C

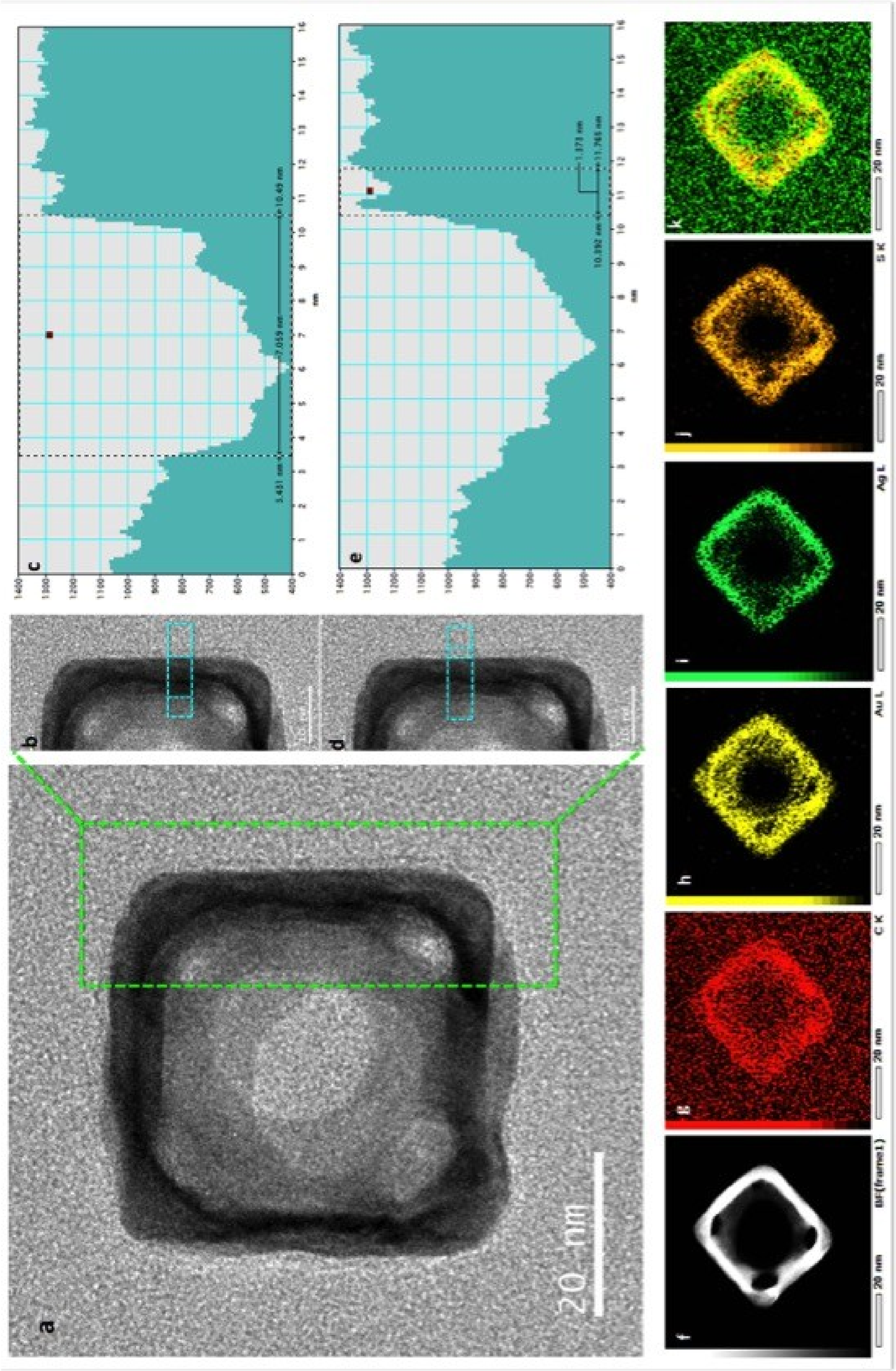
a
20 nm
b
c
d
e
f
BF(frame1)
g
C K
h
Au L
i
Ag L
j
S K
k
20 nm

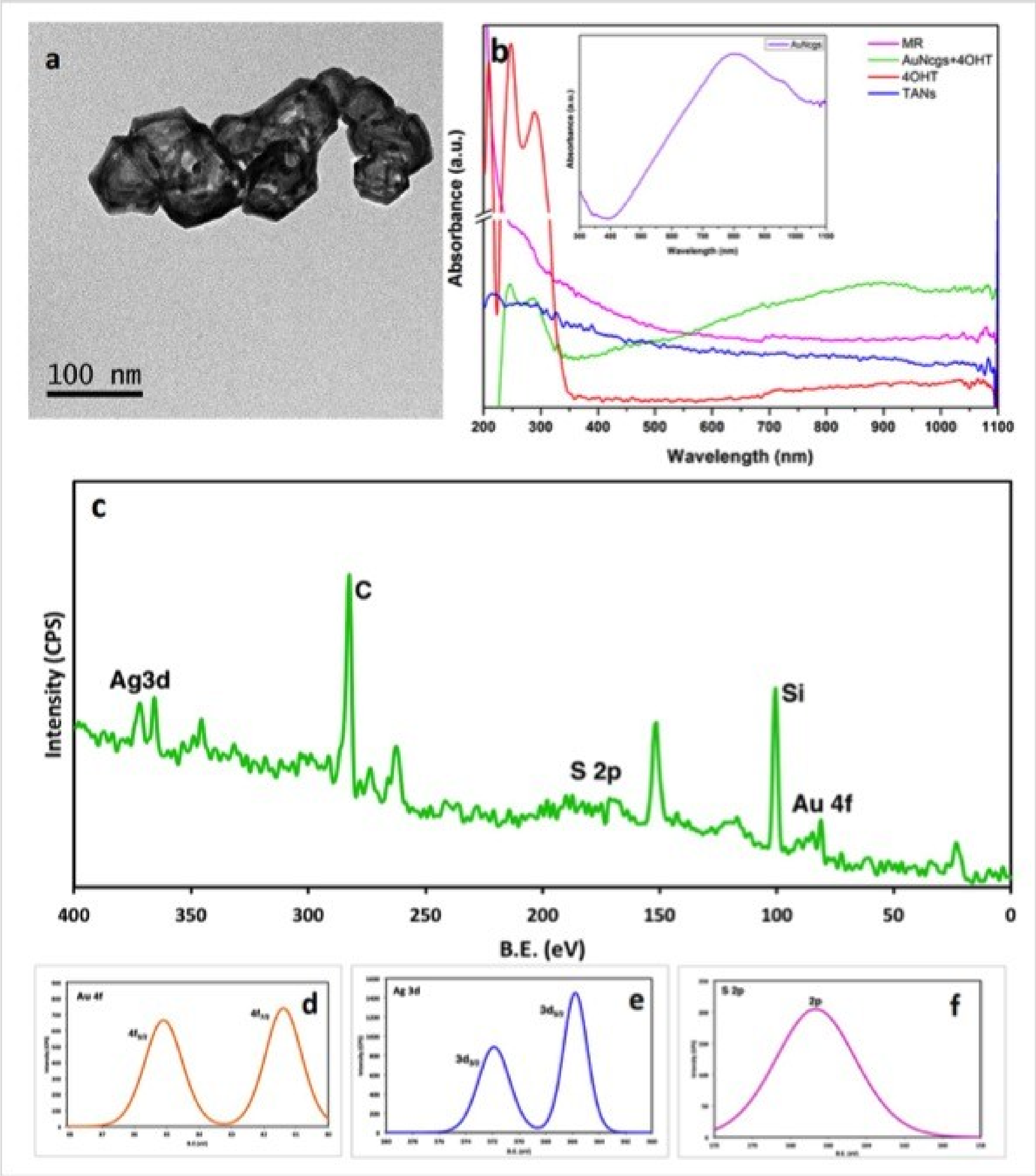
a
100 nm
b
Absorbance (a.u.)
MR
AuNcgs+4OHT
4OHT
TANs
200 300 400 500 600 700 800 900 1000 1100
Wavelength (nm)
c
Intensity (CPS)
C
Ag3d
Si
S 2p
Au 4f
400 350 300 250 200 150 100 50 0
B.E. (eV)
d
e
f

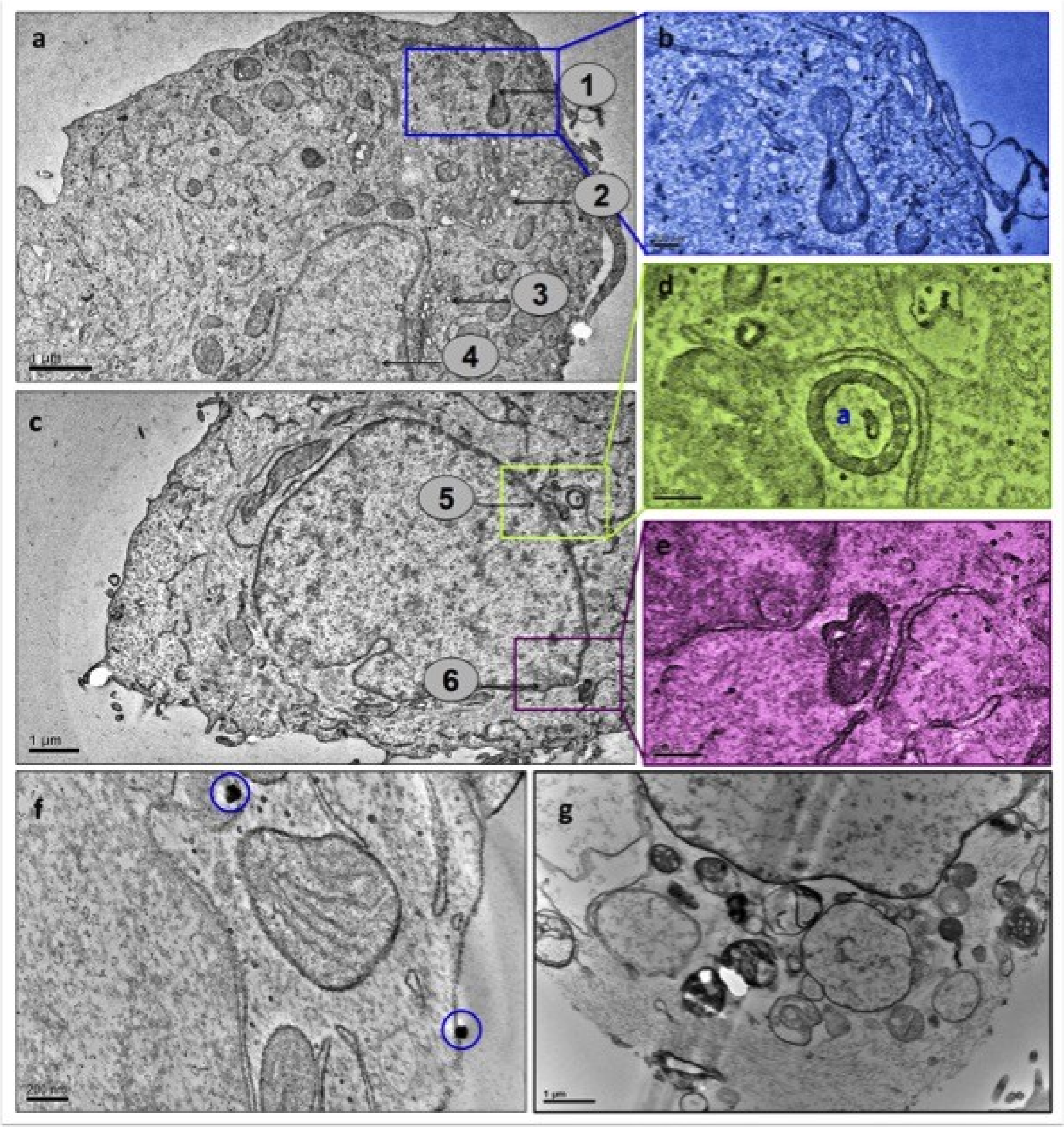
a
1
2
3
4
1 µm
b
c
5
6
1 µm
d
a
e
f
200 nm
g
1 µm

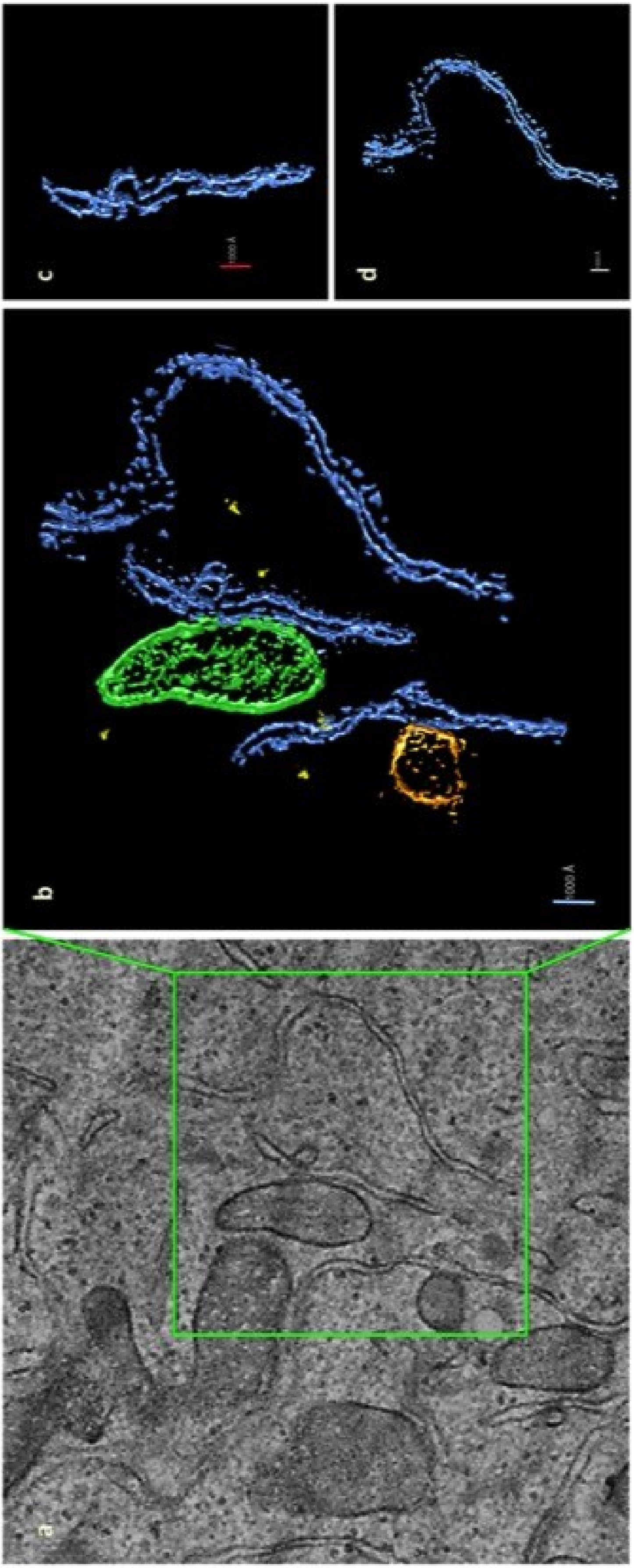
c
1000 Å
d
b
1000 Å
a

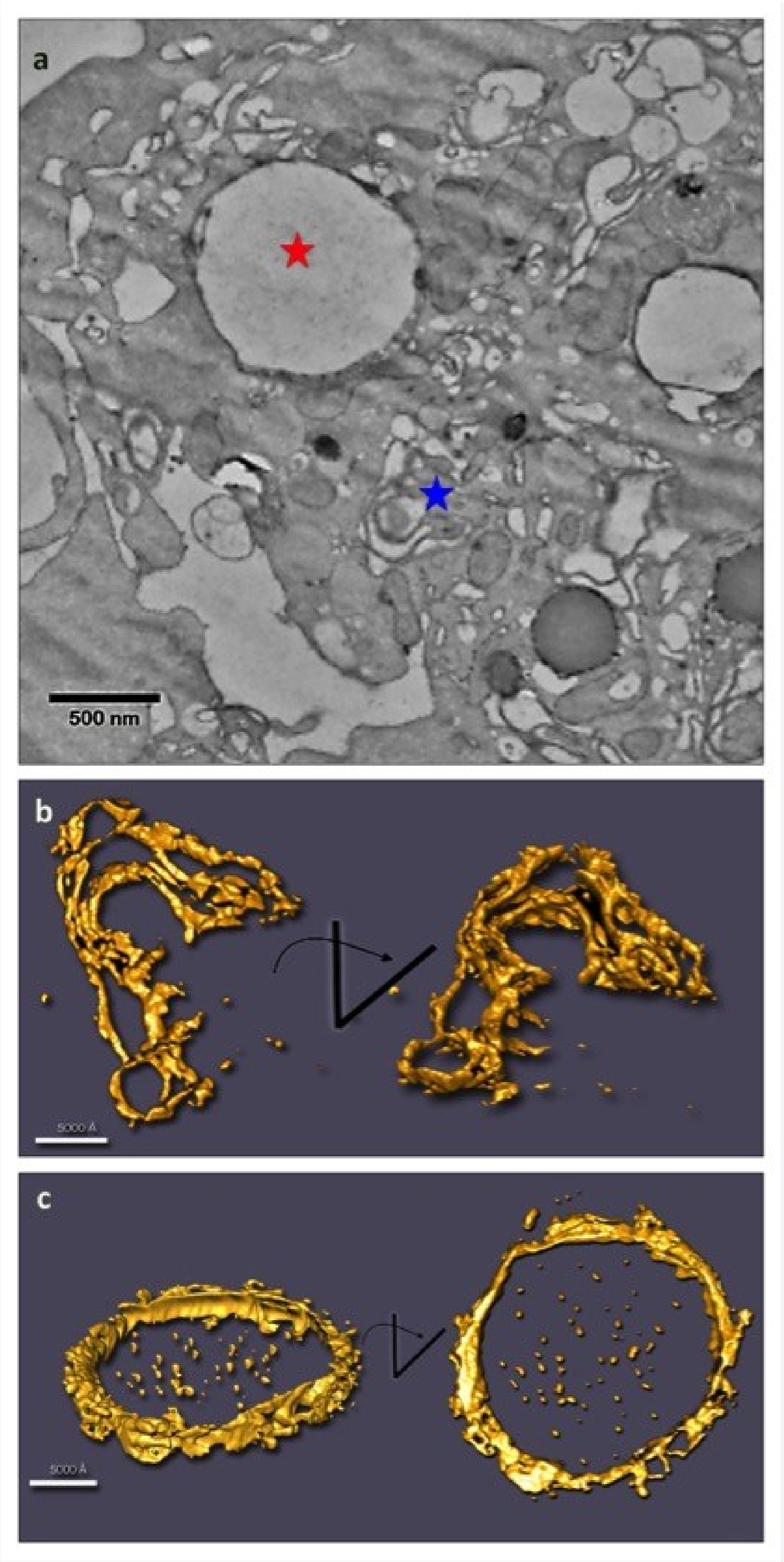
a
500 nm
b
5000 Å
c
5000 Å
